# SOCS2 expression in bone marrow derived dendritic cells is a positive regulator of T cell activation

**DOI:** 10.1186/1479-5876-10-S3-P19

**Published:** 2012-11-28

**Authors:** Jin Hu, Berit Carow, Ann-Charlotte Wikström, Martin Rottenberg, Gunnar Norstedt, Ola Winqvist

**Affiliations:** 1 Dept. of Medicine, Translational Immunology Unit, Karolinska Institutet, Stockholm, Sweden; 2 Dept. of Molecular Medicine and Surgery, Karolinska Institutet, Stockholm, Sweden; 3 Dept. of Biosciences and Nutrition, Karolinska Institutet, Stockholm, Sweden; 4 Dept. of Microbiology, Tumor and Cell Biology, Karolinska Institutet, Stockholm, Sweden

## Background

After a completed T cell response the activation of DCs needs to be terminated to avoid harmful inflammation or autoimmune disease. Besides the negative regulation of JAK/STAT signaling pathway on growth hormone and prolactin for suppressor of cytokine signaling (SOCS) 2 [1], murine SOCS2-/- DCs are recently found to be hyper-responsive to microbial stimuli and refractory to the inhibitory actions of the anti-inflammatory mediator LXA4 [2]. Thus, we investigate the role of SOCS2 in DC antigen presentation.

## Materials and methods

**Mice:** SOCS2 deficient mice and transgenic OT-II mice. **Mouse bone marrow-derived dendritic cells (BMDCs):** Mouse bone marrow cells were incubated 7 days with 20 ng/ml GM-CSF and 20 ng/ml IL-4 to create mouse BMDCs. **Quantitative RT-PCR:** Real-time PCR was used to measure pro-inflamatory cytokines gene expression. **CFSE proliferation assay:** BMDCs were incubated with 100 ng/ml LPS and 50 ng/ml OVA323-339 peptide. The next day CD90+ splenocytes from OT-II mice were labeled with CFSE and added to the BMDC culture for 5 days. Then cells were stained for CD4 and read by FACS.

## Results

**Figure 1:**
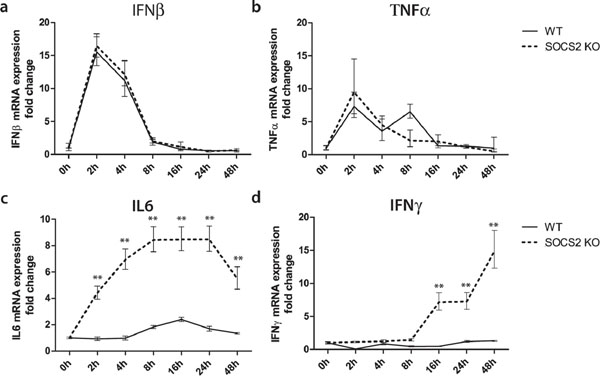
**The effect of SOCS2 KO, on pro-inflammatory cytokine release in LPS induced BMDCs.** BMDCs from WT and SOCS2 mice were stimulated with LPS (100 ng/ml) till 48 hours. mRNA expression of indicated cytokines was measured at the indicated time points.

*Increased production of pro-inflammatory cytokines by SOCS2^-/-^ BMDCs in response to LPS *(see Figure 1)

**Figure 2:**
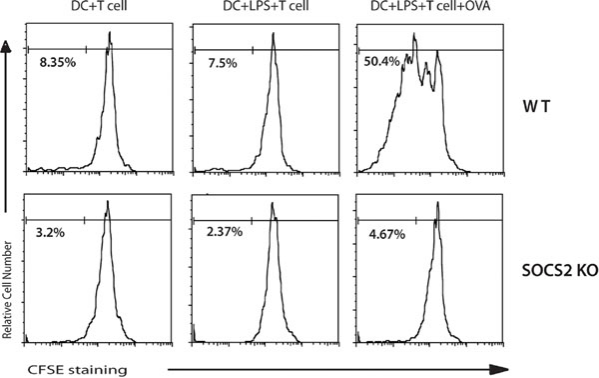
**Proliferation of CD4+ T antigen specific cells is decreased when co-cultured with SOCS2-/- mice BMDCs** The CFSE-labeled OTII CD4^+^ T cells were co- cultured with DCs alone, LPS matured DCs, or LPS matured DCs plus OVA from WT and SOCS2 mice, respectively. The CD4 Tcells were harvested to determine the number of divisions by flow cytometry after 5 days.

*SOCS^-/-^ BMDCs have a decreased capacity to activate naïve CD4^+^ T cells* (see Figure 2)

## Conclusions

SOCS2 is complex regulator of DC effecter functionality, with an overall positive regulatory function on T cell activation.
